# The Biosafety Evaluation for Crustaceans: A Novel Molluscicide PBQ Using against *Oncomelania hupensis*, the Intermediate Host of *Schistosoma japonica*

**DOI:** 10.3390/tropicalmed7100294

**Published:** 2022-10-11

**Authors:** Qianwen Shi, Liping Duan, Zhiqiang Qin, Weisi Wang, Lu Shen, Xuetao Hua, Ling’e Shen, Jiaqian Cao, Fukang Zhu, Jingzhi Wu, Shizhu Li

**Affiliations:** 1 Suzhou Center for Disease Prevention and Control, Suzhou 215004, China; 2 National Institute of Parasitic Diseases, Chinese Center for Disease Control and Prevention (Chinese Center for Tropical Diseases Research), National Health Commission Key Laboratory of Parasite and Vector Biology, WHO Collaborating Center for Tropical Diseases, National Center for International Research on Tropical Diseases, Shanghai 200025, China; 3 Wuzhong District Center for Disease Control and Prevention, Suzhou 215100, China

**Keywords:** *Oncomelania hupensis*, molluscicide PBQ, biosafety evaluation, crustaceans

## Abstract

A new formulation (suspension concentrate, SC) of PBQ [1-(4-chlorophenyl)-3-(pyridin-3-yl) urea] was used in water network schistosomiasis-endemic areas to test its molluscicidal efficacy and the acute toxicity to crustaceans. PBQ (20% SC), 26% metaldehyde, and niclosamide suspension concentrate [MNSC (26% SC)] were used both in ditch and field experiments for the molluscicidal efficacy comparison. Acute toxicity tests of two molluscicides were conducted using *Neocaridina denticulate* and *Eriocheir sinensis*. Both in the field and ditch experiments, PBQ exhibited comparable molluscicidal efficacy with MNSC. At doses of 0.50 g/m^3^ and 0.50 g/m^2^, the snail mortalities were more than 90% three days after PBQ (20% SC) application. Compared with previous tests, PBQ (20% SC) exhibited higher molluscicidal activity than PBQ (25% wettable powder, 25% WP) used in Jiangling and showed similar mollucicidal activity to PBQ (25% WP) used in Dali and Poyang Lake. The 96 h LC_50_ value of MNSC against *Eriocheir sinensis* was 283.84 mg a.i./L. At the concentration of PBQ (20% SC) 1000 mg a.i./L, all *Eriocheir sinensis* were alive. The 96 h LC_50_ values of PBQ and MNSC against *Neocaridina denticulate* were 17.67 and 14.05 mg a.i./L, respectively. In conclusion, PBQ (20% SC) had a comparable molluscicidal efficacy with MNSC (26% SC) and PBQ (25% WP). Furthermore, it showed lower toxicity to the crustacean species, better solubility, no floating dust, and convenience for carriage. PBQ (20% SC) was suitable for controlling snails in the water network schistosomiasis-endemic areas.

## 1. Introduction

Snail-borne parasitic diseases (SBPDs) are major parasitic diseases that remain important public health issues worldwide [1]. SBPDs currently known include schistosomiasis, angiostrongyliasis, fasciolopsiasis, clonorchiasis, opisthorchiasis, paragonimiasis, and so on [1,2]. Parasitic larvae complete the life cycle in freshwater snails, and people can be infected with parasitic diseases by contacting infected water or eating raw, freshwater snails [2].

*Schistosomiasis japonica* (*S. japonicum*), a zoonotic parasitic disease caused by infection of the Schistosoma species, seriously endangers human health and socioeconomic development, which is one of the major global public health concerns [3,4]. Estimates place the affected worldwide population for all forms of schistosomiasis at 230 million, with an estimated 700 million at risk [5]. *Oncomelania hupensis* (*O. hupensis*) is the only intermediate host of *S. japonicum* [6]. The distribution of *O. hupensis* snails determines the epidemic range of schistosomiasis [7]. There is a general rule that in the areas where the epidemic situation has rebounded, the density of *O. hupensis* snails will pick up earlier [8]. Controlling the intermediate snail host has proved to be a very effective strategy of controlling *S. japonicum* [9,10,11]. However, since the 21st century, the total area of *O. hupensis* snails in China has always fluctuated between 3.519 billion and 3.863 billion m^2^, and it seems to be very difficult to further compress the area [12]. Niclosamide is the only molluscicide recommended by the World Health Organization (WHO) and has been widely used internationally because of its high efficiency [13]. However, niclosamide and its derivatives possess high toxicity to fish and other non-target biota, which hinders its applications in areas that are full of aquatic organisms [7,10]. Research and development of high-efficiency, ecofriendly, and cost-effective molluscicides is urgent.

A novel molluscicide PBQ [1-(4-chlorophenyl)-3-(pyridin-3-yl)urea], developed by the National Institute of Parasitic Diseases, Chinese Center for Disease Control and Prevention (Shanghai, China), exhibited strong molluscicidal potency against *biomphalaria straminea* (*B. straminea*) [14], *Pomacea canaliculate* (*P. canaliculata*) [15], and *O. hupensis* [16]. The exhibited molluscicidal potency of PBQ was similar to the niclosamide derivatives widely used now [14,16]. In addition, PBQ showed low toxicity to fish, *Daphnia magna,* and bees [14,15,16], suggesting its relative safety to non-target organisms. Due to the short development time of PBQ, there were few studies. These studies demonstrated the molluscicidal potency of PBQ to *O. hupensis* in hilly and marshland schistosomiasis-epidemic areas without testing its toxicity to crustaceans [17,18]. Moreover, the formulations of PBQ used previously were wettable powders (WP) and floating granules (FG). Wettable powders and floating granules had flying dust during field application, which might harm the health of the users, and both of them were hard to dissolve. Suspension concentrate may avoid these deficiencies and be more suitable for water network schistosomiasis-endemic areas.

In this study, a new dosage form (suspension concentrate, SC) of PBQ was used to test its molluscicidal efficacy and the acute toxicity to crustaceans to provide scientific basis and the rational use method for field promotion in the water network schistosomiasis-endemic areas.

## 2. Materials and Methods

### 2.1. Chemicals

The 20% PBQ sulfate suspension concentrate [PBQ (20% SC)] was provided by the National Institute of Parasitic Diseases, Chinese Center for Disease Control and Prevention (Shanghai, China). The 26% metaldehyde and niclosamide suspension concentrate [MNSC (26% SC)], provided by the Jiangsu Institute of Parasitic Diseases (Wuxi, China), was purchased from Jiangsu Aijin Agrochemical Co., Ltd. (Nanjing, China).

### 2.2. Ditch Experiments

Ditch experiments were conducted in Guangfu Town, Wuzhong District, Suzhou City, Jiangsu Province, China, in June 2022. Seven rectangle-shaped ditches (12.5 m^2^/ditch, 12.5 m length × 1 m width) were prepared for the experiments. Each ditch was enclosed with 0.6 m high soil dikes covered with plastic film to avoid water exchange and the contamination of other treatments. Water was introduced and maintained at a depth of ~40 cm. In each ditch, a total of 154–172 *O. hupensis* (5–7 mm shell length × 3–4 mm shell width) were uniformly divided into 3 bags. PBQ (20% SC) was applied to the study ditches at doses of 0.25, 0.50, 1.00, and 2.00 g/m^3^. PBQ (20% SC) was placed at the center of the ditch and stirred with long sticks to ensure homogeneous mixing. At 3 days after molluscicide application, one bag in each ditch was collected daily, and the mortality of snails was evaluated via the warm water method combined with the percussion method. The arrangement included negative (water) and positive [MNSC (26% SC), 2.00 and 4.00 g/m^3^] controls. Corrected mortality rate of snails was calculated as follows:

Corrected Mortality Rate (%) = (mortality rate molluscicide_application_ − mortality rate _control_)/(100 − mortality rate _control_) × 100% (1)

### 2.3. Field Experiments

Field experiments were carried out in six fields heavily infested with *O. hupensis* near the ditch experiments field in June 2022. Each field covered an area of 100 m^2^ and was surrounded by low soil dikes that were 1 m wide. To avoid dilution of the molluscicide, water circulation from the irrigation channels in each field was stopped before the start of the trail. Before molluscicide application, the density of *O. hupensis* in each field was investigated using a square frame (0.11 m^2^), uniformly distributed in the rectangle-shaped fields. PBQ (20% SC) was applied in three fields by the broadcasted application method at doses of 0.50, 1.00, and 2.00 g/m^2^. The arrangement included negative (water) and positive [MNSC (26% SC), 2.00 and 4.00 g/m^2^] controls. At 3 days post application, snails were sampled using the same protocol used for the snail density investigation. The collected snails from each field were identified as dead or alive via the warm water method combined with the percussion method. The warm water method is to put the snails in warm water (20-5 °C) for 15 min and identify if the snails are dead or alive by observing whether the snails are active. Inactive snails are identified as alive or dead by crushing or percussing [2,5]. The percussion method is to place the snail on a flat glass or a hard object and to tap it. If the soft tissue of the snail is seen to shrink, it is a live snail; otherwise it is a dead snail [2,5]. The corrected mortality rate of snails was consistent with previous mortality rates.

### 2.4. Acute Toxicity Test in Crab

The acute toxicity test in crabs was carried out according to the Guidelines on Environmental Safety Assessment for Chemical Pesticides—Part 21: Macro-crustacean Toxicity Test [19]. Crabs (*Eriocheir sinensis*, 12.76 ± 1.54 g) were obtained from the CTI-Safety Evaluation Technical Service Co., Ltd. (Suzhou, China). Crabs were reared in a big plastic box (2 m × 1 m × 0.6 m) containing dechlorinated water (pH 7.66–8.44, water hardness 170 mg/L CaCO_3_) and maintained at 23.1–23.6 °C, with oxygenation under a 14:10 h (H:D) photoperiod. The plastic box was covered by nets to avoid crabs escaping. The crabs were fed corn once a day. The acclimation continued for about 7 days until the natural mortality rate of the crabs was under 5% when the crabs were suitable for the acute toxicity test. We chose healthy, vigorous, consistent-sized crabs for testing, and feeding was stopped 24 h prior to the experiments. The tests were carried out in glass aquariums (*n* = 10 crabs per aquarium) under static conditions. Based on pre-test results, PBQ (20% SC) and MNSC (26% SC) were set in concentration gradients with equal logarithmic intervals for official tests. According to pre-test results, crabs were exposed to MNSC solutions (20 L) at series concentrations of 73, 130, 231, 411 and 731 mg a.i./L and to PBQ solutions (20 L) at a concentration of 1000 mg a.i./L. The control group received an equal amount of dechlorinated water. The experiment was set up in 3 parallel groups. Throughout the 96 h exposure period, the mortality rate and poisoning symptoms of each group was recorded every 24 h. Dead crabs were removed when death was confirmed.

### 2.5. Acute Toxicity Test in Shrimp

The acute toxicity test in shrimp was performed according to the Guidelines on Environmental Safety Assessment for Chemical Pesticides—Part 21: Macro-crustacean Toxicity Test [19]. Shrimp (*Neocaridina denticulate*, 7.5 ± 0.5 mm) and their fodder (*Artemia salina*) were obtained from CTI-Safety Evaluation Technical Service Co., Ltd. (Suzhou, China). Shrimp were reared in an aquarium (600 mm × 400 mm × 400 mm) containing dechlorinated water (pH 7.65–7.99, water hardness 170 mg/L CaCO_3_) and maintained at 22.9–23.6 °C, with oxygenation under a 14:10 h (H:D) photoperiod. The shrimp were fed with *Artemia salina* once a day. The acclimation continued for at least two weeks to make sure the natural mortality rate of shrimp was under 5%, and feeding was stopped 24 h prior to experiments. The tests were carried out in glass flasks (*n* = 10 shrimp per flask) under static conditions. According to pre-test results, shrimp were exposed to PBQ solutions (1 L) at series concentrations of 4, 8, 16, 32, and 64 mg a.i./L and to MNSC solutions (1 L) at series concentrations of 3.9, 6.9, 12, 22, and 39 mg a.i./L. The control group received an equal amount of dechlorinated water. The experiment was set up in 3 parallel groups. Throughout the 96 h exposure period, the mortality rate and poisoning symptoms of each group was recorded every 24 h. Dead shrimp were removed when death was confirmed.

### 2.6. Data Analysis

Heterogeneity between rates was estimated by the chi-square test (*χ*^2^ test) using Statistical Package for Social Science (SPSS) version 26.0 (IBM Corp., Armonk, NY, USA). The 50% lethal concentration (LC_50_) was calculated using the Benchmark Dose Software version 3.1.2 (U.S. EPA, Washington, DC, USA).

## 3. Results

### 3.1. PBQ Molluscicidal Activity in Ditch Experiments

**Figure 1:**
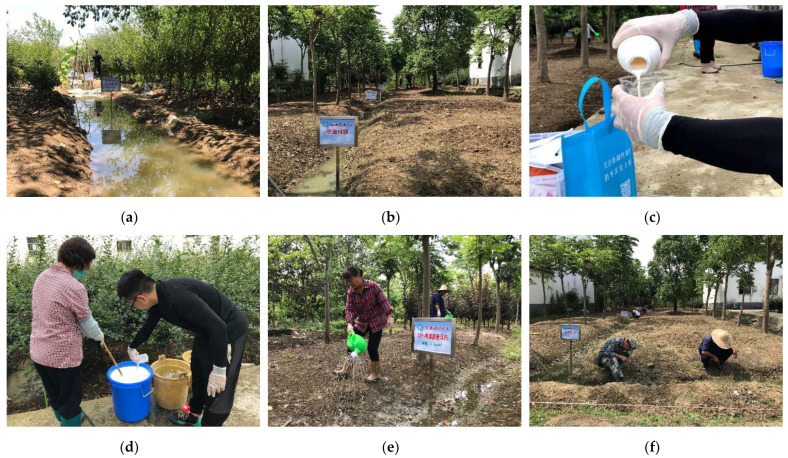

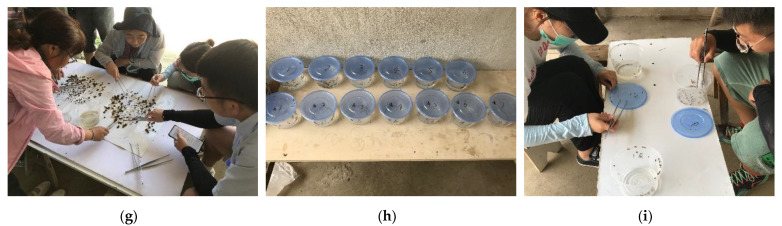
In the ditch experiments, seven rectangle-shaped ditches were prepared for the experiments (**a**). We measured the PBQ and put it into the study ditches (**c**). At 3 days after molluscicide application, snails in each ditch were collected and the mortality of the snails was evaluated via the warm water method combined with the percussion method (**h**,**i**). In the field tests, we first prepared fields for study (**b**). We measured the PBQ (**c**), stirred well (**d**), and sprayed it into the study fields (**e**). Before and after spraying molluscicide, snails were investigated (**f**,**g**) and identified as dead or alive (**h**,**i**).

During the experiments, it was mainly sunny with occasional thundershowers, with the lowest temperature being 26~27 °C, the highest temperature being 32~37 °C, and the air humidity being 66~80%. A 20% suspension concentrate formulation of PBQ was applicated to the *O. hupensis* habitat. The molluscicidal activity of PBQ was first evaluated in ditch experiments (Figure 1).

The snail mortality showed clear dose-dependent effects (*χ*^2^ = 18.353, *p* = 0.003) at 1 day following PBQ treatment. The mortality rates were 70.46, 80.31, 90.53, and 97.54% following PBQ treatment at doses of 0.25, 0.5, 1, and 2 g/m^3^, respectively (Table 1). The highest mortality of *O. hupensis* was at 2 days after PBQ treatment. At the dose of 1 g/m^3^ PBQ, the mortality rate of *O. hupensis* was comparable with the mortality rate at the dose of 4.00 g/m^3^ MNSC after exposure for 1 and 2 days (*χ*^2^ = 0.827, *p* = 0.363; *χ*^2^ = 0.918, *p* = 0.338). After 3 days, the mortality rates of *O. hupensis* were comparable between 2 g/m^3^ PBQ and 4.00 g/m^3^ MNSC (*χ*^2^ = 1.207, *p* = 0.272). At doses equal to or greater than 0.5 g/m^3^, PBQ treatment produced *O. hupensis* snail mortality over 90% at 3 days.

Compared with previous tests, PBQ (20% SC) exhibited higher molluscicidal activity than PBQ (25% wettable powder, 25% WP) used in Jiangling [16] (Table 2). PBQ (20% SC) exhibited similar mollucicidal activity to PBQ (25% WP) used in Dali [18].

### 3.2. PBQ Molluscicidal Activity in Field Trails

The density of *O. hupensis* was 10.2 snails per 0.11 m^2^ in plot experiment areas and field trail experiments. The average natural mortality of *O. hupensis* was 19.6%. During the experiments, it was mainly sunny with occasional thundershowers, with the lowest temperature being 27~28 °C, the highest temperature being 34~38 °C, and the air humidity at 69~83%. The photo of the field trails is shown in Figure 1.

The snail mortality showed clear dose-dependent effects (*χ*^2^ = 38.833, *p* < 0.001) at 1 day following PBQ treatment (Table 3). Meanwhile, the molluscicidal activity of PBQ increased with exposure time. At a dose of 2 g/m^2^ PBQ, the mortality rate of *O. hupensis* was higher than it was at a dose of 2.00 g/m^2^ MNSC (*χ*^2^ = 3.972, *p* = 0.046) and comparable to it at a dose of 4.00 g/m^2^ MNSC after exposure for 1 day (*χ*^2^ = 0.762, *p* = 0.383). There were no statistical differences between the molluscicidal activities of the two molluscicides at 3 and 7 days after exposure (*χ*^2^ = 5.998, *p* = 0.199; *χ*^2^ = 4.110, *p* = 0.391).

Compared with previous tests, PBQ (20% SC) exhibited higher molluscicidal activity than PBQ (25% WP) used in Jiangling [16] and Dali [18] (Table 4). PBQ (20% SC) exhibited similar mollucicidal activity to PBQ (25% WP) used in the marshland around Poyang Lake [17].

### 3.3. Acute Toxicity Test in Crabs

The mortality in the MNSC groups increased with the concentration and the exposure time. No crabs died at a concentration of 130 mg a.i./L of MNSC, but signs of cruising up and down and hypoactivity were observed. The rate and degree of poisoning symptoms increased with the concentration and the exposure time in the MNSC groups. Crabs were all alive when the concentrations of PBQ was 1000 mg a.i./L. The 96 h LC_50_ value of MNSC was 283.84 (260.58–332.26) mg a.i./L (Table 5).

### 3.4. Acute Toxicity Test in Shrimp

For *Neocaridina denticulate*, the mortality in both the PBQ and MNSC treatment groups increased with the concentration and the exposure time. In PBQ treatment groups, shrimp exposed to test concentrations >10 mg a.i./L behaved normally within 24 h, while afterwards, a few individuals exhibited symptoms of poisoning. Initially, their swimming speed declined, then the equilibrium was lost, and finally, some of them died. The same situation occurred when the concentration reached 1 mg a.i./L in MNSC treatment groups. What is more, MNSC exhibited a faster toxicity against *Neocaridina denticulate*. Both at the concentration of 100 mg a.i./L, 90% *Neocaridina denticulate* died in MNSC group while just 10% died in the PBQ group in first 24 h after treatments. The 96 h LC_50_ value of PBQ was 17.67 (14.76–21.04) mg a.i./L and that of MNSC was 14.05 (12.73–15.51) mg a.i./L (Table 5). According to the Guidelines on Environmental Safety Assessment for Chemical Pesticides [19], both PBQ and MNSC were low toxicity [LC_50_ (96 h) > 10 mg a.i./L] chemicals to shrimp.

## 4. Discussion

Schistosomiasis is a public health problem in tropical and subtropical regions of Africa, Asia, the Caribbean, and South America [20]. Last year, the WHO launched a new road map for 2021–2030 that aims to end the suffering from schistosomiasis by 2030 [21]. *O. hupensis* is the only intermediate host of *S. japonicum*, and the control of *O. hupensis* is one of the important methods of the control and prevention of schistosomiasis [5]. Although there are many snail control methods, such as physical, chemical, and biological methods, the cost of physical snail control is high, and there is no effective product for biological snail control [22]. Therefore, killing snails with molluscicide is always the main measure of snail control [23]. However, *O. hupensis* live widely, have an obviously spreading trend, and are difficult to control. Niclosamide demonstrated outstanding molluscicidal activity against different kinds of freshwater snails [24]. However, niclosamide has the disadvantages of poor solubility in water and high toxicity to fish and other non-target biota, which can induce upward climbing movement for snails [10]. Niclosamide can easily cause environmental pollution and contradictions in aquaculture [18]. Therefore, it is important to research and develop novel molluscicides that are environment-friendly and have low toxicity to non-target organisms.

In recent years, the novel molluscicide PBQ showed excellent molluscicidal effects in laboratory experiments and field tests [15]. PBQ has the features of a simple structure, low cost of synthesis, and simple post-processing [14]. PBQ exhibited excellent molluscicidal activity against several kinds of freshwater snails, such as *Pomacea canaliculate* and *Biomphalaria straminea* [14,15,16]. Relatively weaker potency was observed in *O. hupensis*, probably due to its amphibious habitat and thicker shell [16]. Despite this, PBQ showed similar molluscicidal potency to niclosamide and its derivatives widely used today [14,16]. Moreover, PBQ exhibited low toxicity to fish, birds, silkworms, and bees [14,15,16], suggesting its relative safety to non-target organisms. However, its toxicity to crustaceans had not been systematically verified. Since many rice fields are also used as aquaculture systems, such as shrimp or crab farming, and the paddy water containing molluscicides could be discharged into nearby aquaculture ponds, it is particularly important that the molluscicide is low in toxicity to the crustacean species. In this paper, the molluscicidal effect and crustacean biosafety of PBQ and niclosamide were compared and analyzed.

Due to the field tests and ditch experiments in this research, PBQ (20% SC) showed higher molluscicidal activity than MNSC (26% SC) at the same concentration. In field practical application, the mortality rate of *O. hupensis* could be higher than 80% when the concentration of PBQ (20% SC) reached 2.00 g/m^2^ 24 h after exposure, which satisfied the WHO cut-off for the efficacy of a molluscicide formulation [25]. In practice, it could be observed that MNSC (26% SC) yellowed the surrounding plants, while PBQ (20% SC) did not. Compared with previous tests, PBQ (20% SC) exhibited higher molluscicidal activity than PBQ (25% wettable powder, 25% WP) used in Jiangling County, Jingzhou City, Hubei Province, China [16], both in ditch and field tests. PBQ (20% SC) exhibited higher molluscicidal activity than PBQ (25% WP) used in Dali City, Yunnan Province, China in field tests [16,18]. PBQ (20% SC) exhibited similar mollucicidal with PBQ (25% WP) used in the marshland around Poyang Lake [17]. Temperature during experiments in Dali City was 8~24 °C, lower than in this research, which suggested that a molluscicide in a higher temperature range would exhibit better performance. Compared to wettable powder and floating granules, suspension concentrate revealed a better solubility, was more convenient for carriage, and had no floating dust so as to be safe for the operators. In general, the molluscicidal effect of the new dosage form of PBQ was not inferior to that of wettable powder and floating granules and was suitable for water network schistosomiasis-endemic areas. In water network schistosomiasis-endemic areas, the recommended doses of PBQ (20% SC) in ditches and fields were 2 mg/m^3^ and 2 mg/m^2^, respectively.

Due to the acute toxicity test in shrimp, both PBQ (20% SC) and MNSC (26% SC) were low toxicity chemicals to shrimp [19]. However, dead shrimp were found when the concentration of MNSC (26% SC) was 1 mg a.i./L; meanwhile, the concentration of PBQ (20% SC) when the shrimp were found dead was 10 mg a.i./L. The 96 h LC_50_ value of PBQ (20% SC) for shrimp was 17.67 mg a.i./L. PBQ could achieve the ideal effect at concentrations of 2 g/m^2^ and 2 g/m^3^, and the effective concentration corresponding to this concentration was much lower than 17.67 mg a.i./L, indicating that the window of molluscicide is relatively large so as to be used for on-site application in areas around shrimp ponds safely. In addition, PBQ showed low toxicity against shrimp and could provide time for the government sector to deal with it once a leak happens. PBQ (20% SC) seemed much more friendly against crabs. This phenomenon, perhaps related to the tough shell of the crabs, was also observed in other studies [26]. In conclusion, PBQ (20% SC) showed low toxicity to both shrimp and crabs, which is particularly important, since many rice fields are also used as aquaculture systems, such as for fish, shrimp, or crab farming, and the paddy water containing molluscicides could be discharged into nearby aquaculture ponds [15]. Previous studies also showed that PBQ had low acute toxicity to zebrafish and *Daphnia magna* [15]. Because zebrafish, *Daphnia magna*, *Eriocheir sinensis,* and *Neocaridina denticulate* are representative aquatic organisms required for pesticide registration, we could draw the conclusion that PBQ is low in toxicity to aquatic animals.

There are some limitations to this study. In P.R. China, four sub-species of the intermediate host *O.hupensis* have been identified based on morphological and molecular characteristics [27]. We only researched the molluscicidal effect of PBQ (20% SC) on one sub-species of *O.hupensis.* In future studies, we intend to test the molluscicidal effect of PBQ on other sub-species under different terrains, landforms, and latitudes. What is more, this study did not test the molluscicidal effect of PBQ (20% SC) at low concentrations. We also did not research the toxicity of PBQ (20% SC) to aquatic plants. This is the direction we plan to study in the future.

## 5. Conclusions

The molluscicidal activity of PBQ (20% SC) was comparable with MNSC (26% SC). At a dose of 2.00 g/m^2^ PBQ (20% SC), field application could obtain the ideal effect of the controlling of *O. hupensis* snails. Meanwhile, PBQ (20% SC) had a better solubility, convenience for carriage, no floating dust, and low toxicity to crustacean species. PBQ (20% SC) has value for further research and promotion. It could provide novel and effective technical means and tools for controlling snails and advancing the cooperation of schistosomiasis prevention and control between China and Africa.

## Figures and Tables

**Table 1:** Molluscicidal effect of PBQ (20% SC) in ditch experiments.

Molluscicide	Dose (g/m^3^)	D1		D2		D3	
		Mortality (%, n/N) ^a^	Corrected Mortality (%)	Mortality (%, n/N) ^a^	Corrected Mortality (%)	Mortality (%, n/N) ^a^	Corrected Mortality (%)
PBQ (20% SC)	0.25	76.47 (39/51)	70.46	72.58 (45/62)	65.88	88.46 (46/52)	88.46
	0.50	84.31 (43/51)	80.31	100.00 (66/66)	100.00	90.91 (50/55)	90.91
	1.00	92.45 (49/53)	90.53	98.18 (54/55)	97.74	93.33 (56/60)	93.33
	2.00	98.04 (50/51)	97.54	100.00 (52/52)	100.00	98.04 (50/51)	98.04
MNSC (26% SC)	1.00	90.00 (45/50)	87.45	100.00 (55/55)	100.00	100.00 (54/54)	100.00
	2.00	96.43 (54/56)	95.52	100.00 (50/50)	100.00	100.00 (61/61)	100.00
Control	-	20.34 (12/59)	-	19.64 (11/56)	-	24 (12/50)	-

^a^ N, total number of snails; n, number of living snails.

**Table 2:** Molluscicidal effect of different dosage forms of PBQ in ditch experiments.

Study	Molluscicide	Dose (g/m^3^)	D1		D2		D3	
			Mortality (%, n/N) ^a^	Corrected Mortality (%)	Mortality (%, n/N) ^a^	Corrected Mortality (%)	Mortality (%, n/N) ^a^	Corrected Mortality (%)
Suzhou	PBQ (20% SC)	1.00	92.45 (49/53)	90.53	98.18 (54/55)	97.74	93.33 (56/60)	93.33
		2.00	98.04 (50/51)	97.54	100.00 (52/52)	100.00	98.04 (50/51)	98.04
Dali [18]	PBQ (25% WP)	1.00	90.00 (27/30)	89.29	100.00 (30/30)	100.00	100.00 (30/30)	100.00
		2.00	93.33 (28/30)	92.85	100.00 (30/30)	100.00	100.00 (30/30)	100.00
Jiangling [16]	PBQ (25% WP)	1.00	44.23 (23/52)	44.23	80.00 (40/50)	78.70	98.04 (50/51)	98.00
		2.00	34.69 (17/49)	34.69	87.50 (42/48)	86.69	96.08 (49/51)	96.00

^a^ N, total number of snails; n, number of living snails.

**Table 3:** Molluscicidal effect of PBQ (20% SC) in field tests.

Molluscicide	Dose (g/m^2^)	D1		D3		D7	
		Mortality (%, n/N) ^a^	Corrected Mortality (%)	Mortality (%, n/N) ^a^	Corrected Mortality (%)	Mortality (%, n/N) ^a^	Corrected Mortality (%)
PBQ (20% SC)	0.5	60 (48/80)	50.59	97.53 (79/81)	96.59	98.75 (79/80)	98.24
	1	80 (64/80)	75.29	100 (79/79)	100.00	100 (79/79)	100.00
	2	91.36 (74/81)	89.32	97.5 (78/80)	96.55	100 (83/83)	100.00
MNSC (26% SC)	1	80.49 (66/82)	75.90	100 (82/82)	100.00	100 (86/86)	100.00
	2	94.87 (74/78)	93.67	100 (78/78)	100.00	100 (80/80)	100.00
Control	-	19.05 (16/84)	-	27.63 (21/76)	-	29.07 (25/86)	-

^a^ N, total number of snails; n, number of living snails.

**Table 4:** Molluscicidal effect of different dosage forms of PBQ in field tests.

Study	Molluscicide	Dose (g/m^3^)	D1		D2		D3	
			Mortality (%, n/N) ^a^	Corrected Mortality (%)	Mortality (%, n/N) ^a^	Corrected Mortality (%)	Mortality (%, n/N) ^a^	Corrected Mortality (%)
Suzhou	PBQ (20% SC)	1.00	80.00 (64/80)	75.29	100.00 (79/79)	100.00	100.00 (79/79)	100.00
		2.00	91.36 (74/81)	89.32	97.5 (78/80)	96.55	100.00 (83/83)	100.00
Dali [18]	PBQ (25% WP)	1.00	35.48 (11/31)	24.42	57.89 (22/38)	54.71	64.29 (54/84)	58.53
		2.00	66.67 (40/60)	66.67	86.36 (38/44)	86.36	87.39 (97/111)	87.39
Jiangling [16]	PBQ (25% WP)	2.00	21.21 (7/33)	15.37	59.38 (19/32)	58.18	63.33 (19/30)	60.80
Poyang Lake [17]	PBQ (25% WP)	1.00	88.20 (90/102)	87.50	93.10 (95/102)	92.70	90.00 (90/99)	90.40
		2.00	90.90 (90/99)	90.40	95.00 (96/101)	94.70	90.50 (95/105)	90.00

^a^ N, total number of snails; n, number of living snails.

**Table 5:** Acute toxicity of PBQ against *Eriocheir sinensis* and *Neocaridina denticulate*.

Organism	Molluscicide	Time (h)	LC50 (mg a.i./L, 95% CI)
*Eriocheir sinensis*	PBQ (20% SC)	96	>1000
	MNSC (26% SC)	96	283.84 (260.58–332.26)
*Neocaridina denticulate*	PBQ (20% SC)	24	34.20 (27.22–43.79)
		48	25.06 (20.29–31.70)
		72	21.18 (17.19–26.39)
		96	17.67 (14.76–21.04)
	MNSC (26% SC)	96	14.05 (12.73–15.51)

## Data Availability

The datasets used and analyzed during the current study are available from the corresponding authors upon reasonable request.

## References

[1] Lu X.T., Gu Q.Y., Limpanont Y., Song L.G., Wu Z.D., Okanurak K., Lv Z.Y. (2018). Snail-borne parasitic diseases: An update on global epidemiological distribution, transmission interruption and control methods. Infect. Dis. Poverty.

[2] Burton J.B., Clint E.C., Thomas N.O. (2019). Human Parasitology.

[3] Song L.G., Wu X.Y., Sacko M., Wu Z.D. (2016). History of schistosomiasis epidemiology, current status, and challenges in China: On the road to schistosomiasis elimination. Parasitol. Res..

[4] Pisarski K. (2019). The Global Burden of Disease of Zoonotic Parasitic Diseases: Top 5 Contenders for Priority Consideration. Trop. Med. Infect. Dis..

[5] Lackey E.K., Horrall S. (2022). Schistosomiasis. StatPearls.

[6] Liu M.M., Feng Y., Yang K. (2021). Impact of micro-environmental factors on survival, reproduction and distribution of Oncomelania hupensis snails. Infect. Dis. Poverty.

[7] Huang Y.X. (2019). Research and field application of molluscicides in China. Zhongguo Xue Xi Chong Bing Fang Zhi Za Zhi.

[8] Jing X., Shan L., Chun-Li C., Shi-Zhu L., Xiao-Nong Z. (2018). Progress and challenges of schistosomiasis elimination in China. Zhongguo Xue Xi Chong Bing Fang Zhi Za Zhi.

[9] Li Z.J., Ge J., Dai J.R., Wen L.Y., Lin D.D., Madsen H., Zhou X.N., Lv S. (2016). Biology and Control of Snail Intermediate Host of Schistosoma japonicum in The People’s Republic of China. Adv. Parasitol..

[10] Zheng L., Deng L., Zhong Y., Wang Y., Guo W., Fan X. (2021). Molluscicides against the snail-intermediate host of Schistosoma: A review. Parasitol. Res..

[11] Bergquist R., Gray D.J. (2019). Schistosomiasis Elimination: Beginning of the End or a Continued March on a Trodden Path. Trop. Med. Infect. Dis..

[12] Wang T.P., Lu S., Qin Z.Q., Zhou Y.B., Liu Y., Wen L.Y., Guo J.G., Xu J., Li S.Z., Zhang G.M. (2022). Sharing the WHO guideline on control and elimination of human schistosomiasis to achieve the goal of schistosomiasis elimination in China. Zhongguo Xue Xi Chong Bing Fang Zhi Za Zhi.

[13] Sun F., Zhang J.F., Wen L.Y. (2014). Research progress on the molluscicidal effect of niclosamide compounded with other molluscicides against Oncomelania hupensis. Zhongguo Ji Sheng Chong Xue Yu Ji Sheng Chong Bing Za Zhi.

[14] Wang W., Mao Q., Yao J., Yang W., Zhang Q., Lu W., Deng Z., Duan L.J.P. (2018). Discovery of the pyridylphenylureas as novel molluscicides against the invasive snail Biomphalaria straminea, intermediate host of Schistosoma mansoni. Parasites Vectors.

[15] Wang W., Huang S., Liu F., Sun Y., Wang X., Yao J., Li S., Liu Y., Luo B., Zhang X. (2022). Control of the Invasive Agricultural Pest Pomacea canaliculata with a Novel Molluscicide: Efficacy and Safety to Nontarget Species. J. Agric. Food Chem..

[16] Chen Z., Wang W., Yao J., Li S., Zhang X., Hu H., Liu X., Luo B., Liu Y., Xu H. (2019). Toxicity of a molluscicide candidate PPU07 against Oncomelania hupensis (Gredler, 1881) and local fish in field evaluation. Chemosphere.

[17] Zhu Z.-L., Zhang X., Yu Z.-K., Hao Y.-W., Tian T., Wang Q., Duan L.-P., Li S.-Z. (2019). Evaluation on molluscicidal effect of wettable powder of pyriclobenzuron sulphate in marshland schistosomiasis endemic areas. Chin. J. Parasitol. Parasit. Dis..

[18] Bing-Rong L., Wei-Si W., Jun-Min Y., Shi-Zhu L., Hua Y., Jing Y., Shao-Rong C., Yu-Hua L., Li-Ping D. (2019). Molluscicidal activity of 25% wettable powder of pyriclobenzuron sulphate against Oncomelania hupensis robertsoni. Zhongguo Xue Xi Chong Bing Fang Zhi Za Zhi.

[19] (2014). Guidelines on Environmental Safety Assessment for Chemical Pesticides—Part 21: Macro-Crustacean Toxicity Test.

[20] Naidoo P., Mkhize-Kwitshana Z.L. (2022). Clustered Regularly Interspaced Short Palindromic Repeats/ CRISPR associated protein 9-mediated editing of Schistosoma mansoni genes: Identifying genes for immunologically potent drug and vaccine development. Rev. Soc. Bras. Med. Trop..

[21] WHO (2022). WHO Guideline on Control and Elimination of Human Schistosomiasis.

[22] Fenwick A., Rollinson D., Southgate V. (2006). Implementation of human schistosomiasis control: Challenges and prospects. Adv. Parasitol..

[23] Cao C.L., Zhang L.J., Deng W.P., Li Y.L., Lv C., Dai S.M., Feng T., Qin Z.Q., Duan L.P., Zhang H.B. (2020). Contributions and achievements on schistosomiasis control and elimination in China by NIPD-CTDR. Adv. Parasitol..

[24] He P., Wang W., Sanogo B., Zeng X., Sun X., Lv Z., Yuan D., Duan L., Wu Z. (2017). Molluscicidal activity and mechanism of toxicity of a novel salicylanilide ester derivative against Biomphalaria species. Parasites Vectors.

[25] Ayi I., Chandre F., Coelho P., El-Harawy A., Elemam M., Gachuhi K., Jian-Rong D., Kariuki C., Madsen H., Moné H., Yadav R.S. (2019). Guidelines for Laboratory and Field Testing of Molluscicides for Control of Schistosomiasis.

[26] Lu J., Zhang J.J., Wang P.P., Qing H., Zhou G.Q. (2020). Toxicity and Safety Evaluation of Two Pesticides against Red Swamp Crayfish Procambarus clarkii and Chinese Mitten Crab Eriocheir sinensis. Fish. Sci..

[27] Gordon C.A., Kurscheid J., Williams G.M., Clements A.C.A., Li Y., Zhou X.N., Utzinger J., McManus D.P., Gray D.J. (2019). Asian Schistosomiasis: Current Status and Prospects for Control Leading to Elimination. Trop. Med. Infect. Dis..

